# Cytokine Profile in Aqueous Humor of Patients With Ocular Toxocariasis

**DOI:** 10.3389/fmed.2022.869976

**Published:** 2022-05-13

**Authors:** Zhaoxin Jiang, Limei Sun, Xiaohu Ding, Ting Zhang, Songshan Li, Xiaoyan Ding

**Affiliations:** State Key Laboratory of Ophthalmology, Zhongshan Ophthalmic Center, Sun Yat-sen University, Guangzhou, China

**Keywords:** ocular toxocariasis, aqueous humor, cytokine profile, T-helper type, uveitis

## Abstract

**Purpose:**

Ocular toxocariasis (OT) is a vision-threatening disease with a largely unknown intraocular pathogenesis. Herein, we determined the cytokine expression profile in aqueous humor (AH) of patients with OT.

**Methods:**

This is a retrospective case-control study of cytokine levels in AH of patients with OT and uveitis and control subjects. Thirty samples from eyes with OT, 23 from eyes with non-OT uveitis, and 25 from eyes with age-related cataract were analyzed using a multiplexed magnetic bead immunoassay. Thirty-one cytokines were detected and classified into 5 categories: T-helper type 1 (Th1) -associated cytokines, Th2-associated cytokines, Th17 cytokine, proinflammatory mediators, and growth factors.

**Results:**

In the 31 cytokines, 9 cytokines were undetectable, including IL-1a, IL-1b, IL-2, IL-3, IL-12p70, IL-17A, TGF-a, TNF-β, and IFN-g. From the 22 cytokines, 13 exhibited significantly increased expression in the OT group than in the control group, including TNF-a, IFN-a2, IL-4, IL-5, IL-6, IL-9, IL-10, IL-13, sCD40L, PDGF-AA, PDGF-AB/BB, FLT3l, and EGF. There were 5 cytokines exhibited significantly increased expression in the OT group than in non-OT group, including IL-5, IL-9, IL-10, IL-13, and PDGF-AA. There was no significantly decreased expression in any cytokines in the OT group when compared with control or non-OT groups. To the 5 cytokines that showed significant difference in OT group alone, IL-10 and IL-13 exhibited more than 13-fold increase, and IL-5 showed the most obvious as 27-fold increase in OT patients, when compared with that in control group.

**Conclusion:**

The cytokine profile expression in aqueous humor from patients with ocular toxocariasis was investigated, and our findings suggest that Th1 and Th17 cytokine responses are not enhanced, whereas the cytokine status was polarized toward a Th2 response. Our findings also suggest the involvement of IL-5, IL-10, and IL-13 in the immunopathogenesis of ocular toxocariasis.

## Introduction

Ocular toxocariasis (OT) is an intraocular parasitic infection caused by larvae of the roundworm *Toxocara* (1, 2). It is reported to be an important cause of visual impairment during childhood with a mean age of onset of 7.5 years (1, 3). OT is typically unilateral and includes clinical manifestations such as retinal granuloma, comorbidity with uveitis, epiretinal membrane, and retinal detachment. It leads to permanent retinal damage, visual loss, and strabismus (4, 5). Furthermore, the diagnosis and treatment of OT are still challenging. In clinical practice, no signs or symptoms are pathognomonic for OT. Diagnosis is usually based on the combination of fundoscopy, multimodal imaging features, and immunological tests (1, 4).

Despite OT being a well-known disease, intraocular host response and concrete mechanisms remain largely unknown. OT is believed to be derived from the intraocular immune response to the presence of Toxocara larvae and their products (6, 7). Cytokines play an important role in the coordination of the immune response, and their changing profile may unravel the underlying mechanism. Nagy et al. showed increase of protective pro-inflammatory cytokines (IL-6 and IL-13) and anti-inflammatory IL-10 in Toxocara-seropositive children with chronic cough (8). Fan CK revealed significantly elevated levels of IL-4 and IL-5 in the cerebrum of T. canis-induced cerebral toxocariasis in mice (9). To the best of our knowledge, no previous study examined the cytokine expression pattern associated with OT. Therefore, it is important to elucidate the influences of multiple cytokines on the pathogenesis of OT.

Multiplex bead-based Luminex technology provides rapid and quantitative measurement of multiple targets simultaneously (total of 25–50 μL) and are considered more effective and sensitive than traditional Enzyme-linked immunosorbent assay (ELISA) for detecting specific antibodies (10, 11). Thus, it was applied to detect 31 cytokines simultaneously in OT patients’ AH samples, which were collected prior to any treatment, and compared them with those in eyes with non-OT uveitis and control subjects. The study clarified the cytokine profile in OT with a view to determining which cytokines are potential diagnosis biomarkers or treatment targets in the future.

## Materials and Methods

### Patients and Control Subjects

This is a retrospective case-control study of cytokine levels in AH of patients with OT, uveitis and control subjects. AH samples (approximately 100 μL) were collected from patients with uveitis by using a 30-gauge needle. Control AH was collected from patients who were undergoing routine phacoemulsification. All procedures adhered to the tenets of the Declaration of Helsinki, and local approval was received from the Investigational Review Board of Zhongshan Ophthalmic Center, Sun Yat-sen University. Informed consent was obtained from the subjects after explanation of the nature and possible consequences of the study.

Diagnosis of OT was based on the following criteria: (1) the typical and specific manifestations including unilateral chorioretinal granuloma in the peripheral or posterior pole, and diffuse nematode endophthalmitis; (2) positive specific anti-OT IgG levels and a Goldmann-Witmer coefficient higher than 3 in paired AH and serum samples (12); (3) exclusion of other ocular diseases, such as ocular toxoplasmosis, sarcoidosis, ocular tuberculosis, and any other form of infectious uveitis. Patients diagnosed with non-OT uveitis, infectious uveitis of other etiologies, or idiopathic uveitis, were also recruited. Patients with age-related cataracts who underwent routine phacoemulsification surgery were selected as the control group. The exclusion criteria were as follows: (1) history of any other ocular diseases, apart from age-related cataracts; (2) any previous intraocular surgery; and (3) patients with serious systemic diseases, including diabetes, heart, lung, liver, and kidney dysfunction. AH samples were collected when patients were highly suspected of OT diagnosis, and demographic data and ocular characteristics of the patients were recorded.

### Sample Collection

Seventy-eight AH samples from 78 eyes were collected consecutively, including 30 samples from eyes with OT, 23 from eyes with non-OT uveitis, and 25 from eyes with age-related cataract. Sampling of AH was performed under a surgical microscope after sterilizing the surface of the cornea and conjunctiva with povidone-iodine. Approximately 100 μL of AH was collected *via* limbal paracentesis with the use of a 30-gauge needle. Each sample was centrifuged (3,000 rpm for 5 min), separated into cellular component and supernatant components, and frozen at −80°C until use.

### Cytokine Assays

The advent of bead-based multi-detection assays has made possible the measurement and correlation of multiple cytokine analytes from a single, small, AH sample. This technology utilizes internally color-coded magnetic microspheres, coupled to analyte-specific antibodies, allowing for the simultaneous measurement of up to 50 analytes within each sample. A customized bead panel kit which measures 31 cytokines in a 96-well format (Milliplex Human Cytokine/Chemokine Magnetic Bead Panel I kit, Cat. # HCYTMAG-60K; Billerica, MA, United States), was employed in this study (13). These cytokines were classified into 5 categories: (1) T-helper type 1 cytokines: interleukin (IL)-2, IL-7, IL-12p40, IL-12p70, interferon alpha 2 (IFN-α2), IFN-γ, tumor necrosis factor alpha (TNF-α), TNF-β, and IL-15; (2) T-helper type 2 cytokines: IL-3, IL-4, IL-5, IL-9, IL-10, and IL-13; (3) T-helper (Th) 17 cytokine: IL-17A; (4) proinflammatory mediators: interleukin-1 receptor antagonist (IL-1ra), IL-1α, IL-1β, IL-6, IL-8, and soluble CD40 ligand (sCD40L); and (5) growth factors: granulocyte-colony stimulating factor (GCSF), granulocyte-macrophage colony-stimulating factor (GM-CSF), epidermal growth factor (EGF), fibroblast growth factor 2 (FGF-2), fms-like tyrosine kinase 3 ligand (FLT3l), transforming growth factor alpha (TGF-α), platelet-derived growth factor composed of 2 A subunits (PDGF-AA), PDGF-AB/BB, and vascular endothelial growth factor (VEGF) (14, 15).

The assay was performed according to the manufacturer’s instructions. Undiluted samples (25 μL neat per well) were assayed in duplicate. Standard curves for each cytokine were generated from reference standards supplied with the kit. Data were analyzed by Luminex FlexMap 3D (Luminex, Austin, TX, United States). Cytokine concentrations were determined by Luminex Xponent 4.2 using 5-p log analysis. Concentrations above or below the detection limit were given as the highest or lowest detectable value. For statistical analysis, concentrations below the detection limit were converted to a value of 0.5 × the lowest point on the calibration curve.

### Statistical Analyses

Normally distributed data were determined by the Shapiro-Wilk test and expressed as mean ± standard deviation, while non-normal data were expressed as median and range, because outliers were present. Kruskal–Wallis test was used for more than two groups comparison (OT, non-OT uveitis and controls), and Wilcoxon–Mann–Whitney rank sum test was used for the two-group comparison. Bonferroni correction was used in pairwise comparisons. Venn diagram was generated by using Venny 2.1 (available in the public domain at https://bioinfogp.cnb.csic.es/tools/venny/index.html). Forest diagram was generated by using Excel 2020 (Microsoft). Heatmaps were generated using the heatmap.2 function in the GPLOTS 2.11.0 library for R version 2.15.0 (available in the public domain at http://cran.us.r-project.org; Comprehensive R Archive Network) on log2-transformed values of cytokine concentrations for all samples in each groups, to provide an overall view of the changes in cytokine values for all patients. For all statistical analyses, *P* < 0.05 was considered statistically significant.

## Results

Using a multiplexed magnetic bead immunoassay, we measured the levels of cytokines in AH samples: 30 from OT affected eyes, 23 from eyes affected with non-OT uveitis, and 25 control cases. The mean ages of the patients were 12.3 ± 3.43, 20.7 ± 4.29, and 75.5 ± 9.27 years in OT, non-OT uveitis, and control groups, respectively (*p* = 0.016). The male/female ratio was equal in three groups (*P* = 0.658). In non-OT uveitis group, there were 16 cases of non-infectious uveitis (6 Behcet’s disease, 4 idiopathic uveitis, 3 cases of Vogt–Koyanagi–Harada, 2 ankylosing spondylitis, and 1 sarcoidosis) and 7 cases of infectious uveitis (3 ocular tuberculosis, 2 herpetic anterior uveitis, and 2 toxoplasma). In OT patients, retinal granulomas (28 cases, 93.33%), retrolental membranes (21 cases, 70.0%) and branch-like vitreous strands (16 cases, 53.33%) were most common clinical features. The clinical features above were found in only 5 cases (21.74%), 3 cases (13.04%), and none of 23 patients in non-OT uveitis group, respectively. From the 31 cytokines, 9 cytokines were undetectable, including IL-1a, IL-1b, IL-2, IL-3, IL-12p70, IL-17A, TGF-a, TNF-β, IFN-g. A complete dataset of cytokine concentration is summarized in Table 1.

**TABLE 1:** Summary of cytokines measured by multiplex bead immunoassay (ng/ml).

Categories	Cytokines	OT median (Q25, Q75)	Non-OT uveitis median (Q25, Q75)	Control median (Q25, Q75)	*P*-value (K_W test)	*Adj. P*-value OT vs. control	*Adj. P*-value OT vs. uveitis	*Adj. P*-value uveitis vs. control
T helper type 1	TNF-α	2.04 (0.85, 3.93)	0.85 (0.85, 1.88)	0.85 (0.85, 0.85)	<*0.001*	*0.003*	*0.237*	*0.021*
	IFN-α2	6.86 (3.45, 13.93)	5.99 (3.45, 11.28)	5.12 (3.45, 5.12)	*0.025*	*0.015*	*1.878*	0.309
	IFN-γ	1.39 (1.39, 1.39)	1.39 (1.39, 3.67)	1.39 (1.39, 1.39)	*0.027*	*0.183*	*0.711*	*0.021*
	IL-2	0.74 (0.74, 0.74)	0.74 (0.74, 0.74)	0.74 (0.74, 0.74)	0.104			
	IL-12p70	1.50 (1.50, 1.50)	1.50 (1.50, 1.50)	1.50 (1.50, 1.50)	0.168			
	IL-7	2.34 (1.00, 3.77)	2.02 (1.00, 2.66)	2.66 (2.34, 2.98)	0.198			
	TNF-β	1.32 (1.32, 1.32)	1.32 (1.32, 1.32)	1.32 (1.32, 1.32)	0.449			
	IL-12p40	6.59 (4.33, 11.99)	6.58 (3.83, 12.57)	5.87 (4.16, 7.49)	0.450			
	IL-15	3.29 (2.21, 5.81)	3.18 (0.83, 7.01)	2.98 (2.31, 3.59)	0.543			
T helper type 2	IL-10	26.18 (5.30, 98.98)	3.19 (1.31, 20.77)	1.31 (1.31, 1.31)	<*0.001*	*0.003*	*0.036*	*0.003*
	IL-13	14.11 (2.09, 42.70)	1.31 (0.32, 2.36)	1.07 (0.84, 1.31)	<*0.001*	*0.003*	*0.003*	1.059
	IL-9	2.88 (1.18, 7.71)	1.18 (1.18, 1.18)	1.18 (1.18, 1.18)	< *0.001*	*0.003*	*0.003*	3.000
	IL-5	39.58 (6.61, 78.53)	1.47 (1.47, 1.47)	1.47 (1.47, 1.47)	<*0.001*	*0.003*	*0.003*	0.891
	IL-4	9.72 (6.29, 21.38)	9.72 (2.40, 13.43)	6.29 (6.29, 9.72)	*0.010*	*0.006*	0.654	0.432
	IL-3	0.99 (0.99, 0.99)	0.99 (0.99, 0.99)	0.99 (0.99, 0.99)	1.000			
T helper (Th) 17 cytokine	IL-17A	1.54 (1.54, 0.54)	1.54 (1.54, 1.54)	1.54 (1.54, 1.54)	1.000			
Proinflammatory mediators	sCD40L	3.48 (1.53, 7.71)	1.53 (1.53, 4.87)	1.53 (1.53, 1.53)	<*0.001*	*0.003*	0.684	*0.015*
	IL-6	55.65 (25.03, 151.69)	49.58 (1.33, 168.77)	4.36 (1.33, 20.52)	<*0.001*	*0.003*	0.774	*0.009*
	IL-1a	1.18 (1.18, 2.49)	1.18 (1.18, 4.17)	1.18 (1.18, 1.18)	*0.090*			
	IL-8	17.60 (6.87, 58.33)	8.58 (1.32, 38.23)	8.88 (6.27, 10.59)	0.085			
	IL-1ra	20.03 (9.54, 33.72)	13.30 (5.95, 24.56)	12.21 (10.06, 15.52)	0.267			
	IL-1b	1.34 (1.34, 1.34)	1.34 (1.34, 1.34)	1.34 (1.34, 1.34)	0.303			
Growth factors	PDGF-AA	48.42 (31.52, 66.53)	22.80 (8.97, 44.57)	22.76 (20.25, 27.35)	<*0.001*	*0.003*	*0.003*	2.925
	PDGF-AB/BB	3.72 (0, 8.08)	1.48 (1.48, 12.12)	1.48 (1.48, 1.48)	<*0.001*	*0.003*	1.911	*0.003*
	FLT3l	30.06 (21.64, 47.92)	18.43 (9.88, 47.25)	11.00 (7.52, 12.08)	<*0.001*	*0.003*	0.246	*0.015*
	GCSF	23.17 (11.52, 55.24)	16.02 (4.57, 25.48)	14.39 (11.93, 16.83)	*0.030*	*0.051*	*0.114*	2.901
	EGF	5.20 (3.89, 5.82)	4.55 (3.89, 5.82)	3.89 (3.89, 4.55)	*0.035*	*0.018*	0.987	0.840
	VEGF	32.80 (11.13, 71.45)	28.96 (14.83, 50.22)	58.81 (34.95, 75.00)	*0.050*	0.177	2.424	*0.054*
	GM-CSF	6.54(4.46, 10.87)	4.93 (2.38, 7.69)	4.74 (4.08, 5.51)	0.050			
	TGF-α	1.17 (1.17, 1.17)	1.17 (1.17, 1.17)	1.17 (1.17, 1.17)	0.294			
	FGF-2	24.66 (19.36, 33.72)	24.66 (19.36, 44.87)	24.66 (24.66, 31.56)	0.956			

Bonferroni correction was used in pairwise comparisons. Adj. P-value = P*3, when Adj. p-value was less than 0.05, differences between groups were considered statistically significant.

**FIGURE 1:**
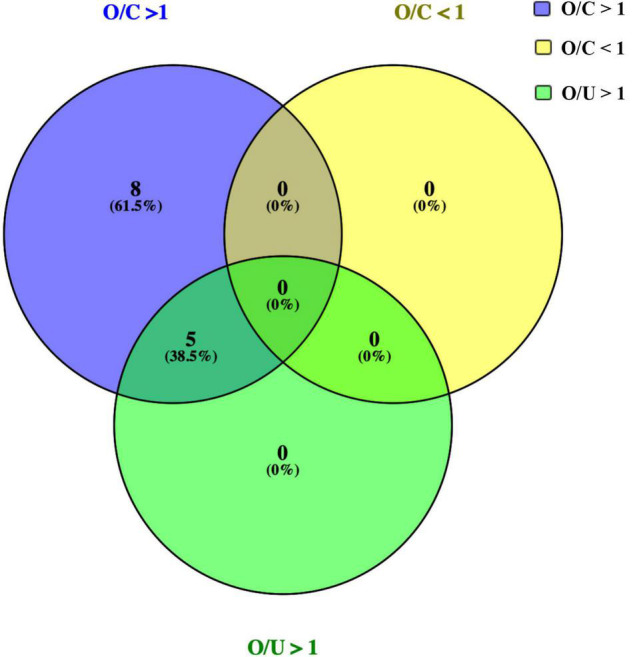
Venn diagram showing the numbers and overlap of cytokines analyzed in the 3 groups. Expressions of 13 cytokines were increased significantly in the OT group than those in the control group (O/C > 1), including TNF-a, IFN-a2, IL-4, IL-5, IL-6, IL-9, IL-10, IL-13, sCD40L, PDGF-AA, PDGF-AB/BB, FLT3l, and EGF. Among them, 5 cytokines, including IL-5, IL-9, IL-10, IL-13, and PDGF-AA, were increased significantly in OT group, if compared with those in non-OT group (O/U > 1). No significant decrease of cytokines was identified in OT group, when compared with those in control/non-OT groups.

To analyze the changing trend of cytokines, we depicted a Venn diagram (Figure 1). From the 22 cytokines, 13 exhibited significantly increased expression in the OT group than in the control group, including TNF-a, IFN-a2, IL-4, IL-5, IL-6, IL-9, IL-10, IL-13, sCD40L, PDGF-AA, PDGF-AB/BB, FLT3l, and EGF. There were 5 cytokines exhibited significantly increased expression in the OT group than in non-OT group, including IL-5, IL-9, IL-10, IL-13, and PDGF-AA. There was no significantly decreased expression in any cytokines in the OT group when compared with control or non-OT groups.

**FIGURE 2:**
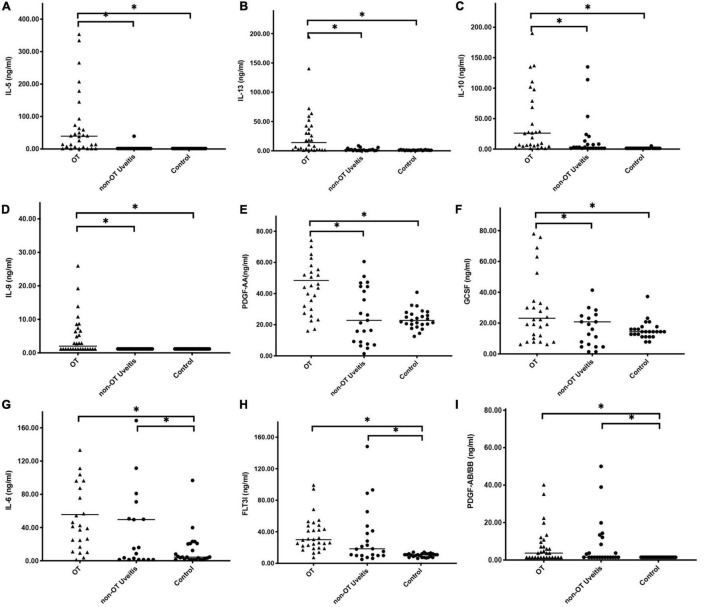
Scattered distribution of representative cytokines concentration among three groups. Between 31 cytokines, expressions of IL-5, IL-10, IL-13, IL-9, and PDGF-AA were significantly increased in OT group alone, when compared with uveitis and control group **(A–F)**. IL-6, FLT3L, and PDGF-BB also showed significant differences between OT and control groups **(G–I)**, but there were no significant differences between OT and uveitis groups. *P < 0.05.

Next, scattered distribution of cytokines concentration among three groups were investigated (Figure 2). Deviations were observed in all cytokine expression profiles; however, a number of significant differences were still detected based on current data. Results revealed that levels of IL-6, IL-10, TNF-α, FLT3l, PDGF-AB/BB, and sCD40L were significantly increased in both OT and non-OT uveitis patients, compared with the control group levels. Moreover, expression of IL-5, IL-9, IL-10, IL-13, and PDGF-AA, were significantly increased in the OT group alone, compared with the non-OT uveitis and control groups.

**FIGURE 3:**
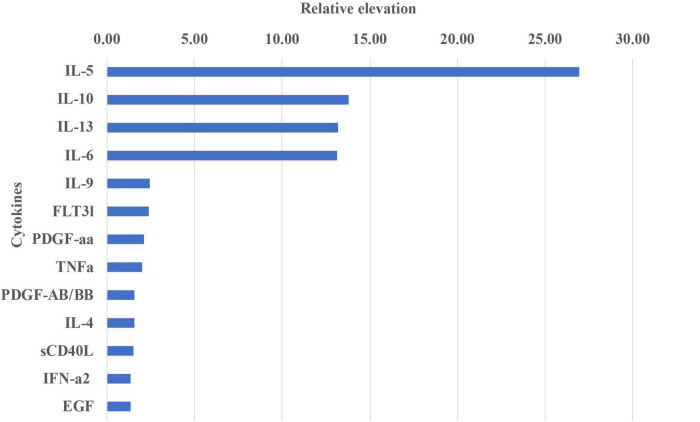
The relative importance of individual cytokines changed in aqueous humor between OT and control groups. The vertical axis shows the individual cytokines with significant difference between OT and control group. The horizontal axis represents the average increase fold of expression levels. IL-5 showed the most noticeable expression increase, followed by IL-10, IL-13, and IL-6, which showed 13-folds or more difference between the groups.

Furthermore, the extent of expression changes among the three groups was explored *via* forest analysis (Figure 3). Among the cytokines that showed significant difference (*P* < 0.05), IL-5 showed the most obvious, 27-fold increase compared with that in controls. Furthermore, IL-6, IL-10, and IL-13 exhibited more than 13-fold increase in OT patients compared with that in controls. IL-5, IL-10, and IL-13 showed 3-fold or more significant elevations in OT than in non-OT uveitis patients.

**FIGURE 4:**
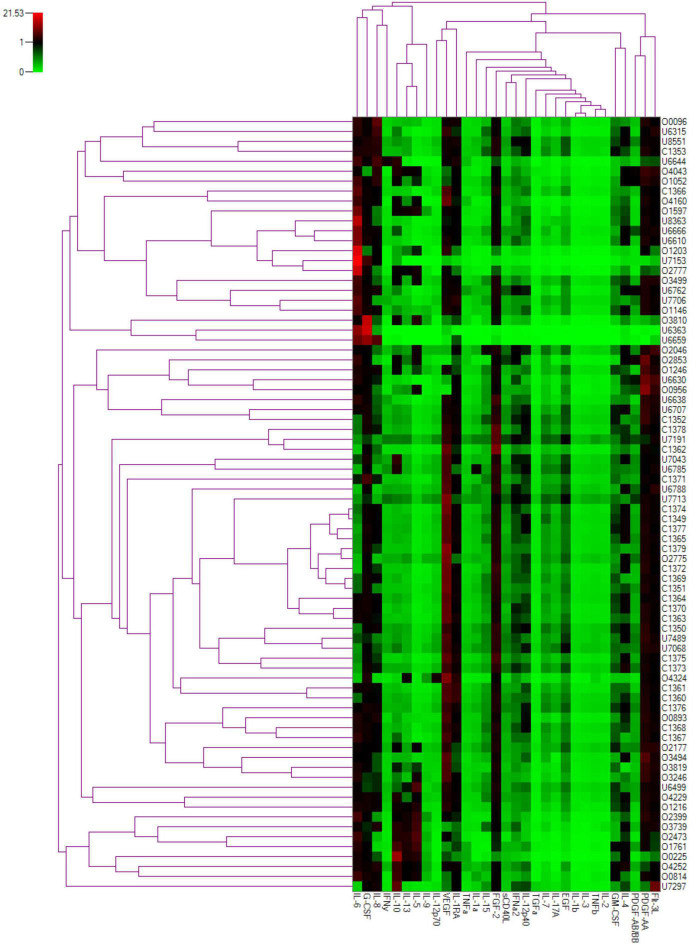
Two-dimensional cluster analysis of the cytokine dataset. Expression levels of individual cytokines are represented by shades of green to red in the central heatmap, with highest values in bright red and the lowest in light green. The similarity between the profiles of different patients is represented by the left dendrogram, whereas the relationship between cytokines is represented in the dendrogram on the top.

We also reviewed the cytokines based on their role in inflammation. Heat-map analysis revealed that the cytokines were broadly clustered into two groups, as seen on the top dendrogram in Figure 4, based on the overall cytokine levels in all patients. The first cluster at left end of the dendrogram consisted of IL-5, IL-9, IL-10, and IL-13, the expression pattern of which were consistent with statistical results as shown in Table 1. The second cluster at right, including IL-1β, IL-2, IL-3, and TNF-β, had low levels of expression in all samples.

Thus, most Th1 cytokines, including IL-2, IL-7, IL-12p40, IL-12p70, IL-15, and TNF-β, were unchanged between the OT and control groups. The most significant increase was detected in Th2 cytokines, including IL-4, IL-5, IL-9, IL-10, and IL-13. There was no change in Th17 cytokine levels, represented by IL-17. Proinflammatory mediators showed significant changes in IL-1α, IL-6, and sCD40L levels, but no difference in IL-1ra, IL-1β, and IL-8. Growth factors, including PDGF-AA, PDGF-BB, EGF, and FLT3l, also showed significant level difference, but not in VEGF, GCSF, GM-CSF, TGF-α, and FGF-2.

## Discussion

Toxocariasis is one of a group of diseases known as neglected parasitic infections, targeted by the Centers for Disease Control and Prevention for public health action (16, 17). This disease is classified as neglected because relatively little attention has been directed toward its surveillance, prevention and treatment (4). Clinically, OT shares certain clinical features with uveitis of other etiology (18, 19), while diagnosis of OT is hindered by the atypical clinical signs and the limited knowledge of the physicians. Thus, specific cytokines profile may help the diagnosis greatly. In this study, these expressions were analyzed in OT patients and compared with the non-OT uveitis and control groups. Collectively, significant increase was detected in Th2 cytokines level mostly, as well as growth factors and proinflammatory mediators, but only a slight increase in Th1 cytokines. This profile is expected to help understand the pathogenesis of OT, and ultimately may help differentiate OT from uveitis caused by other factors.

Our results showed that Th2 cells may be the major effector T cells in OT. CD4 + T cells are differentiated into two functional subsets based on their profiles of cytokine production. Th1 cells produce IL-2, IL-7, IL-12p40, IL-15, and TNF-α. They are responsible for cell-mediated immunity, and they are also involved in the pathogenesis of organ-specific autoimmune disorders (20). In contrast, Th2 cells, which produce IL-4, IL-5, IL-6, IL-9, IL-10, and IL-13, induce strong antibody responses by B cells, induce eosinophil activation, and are responsible for allergic reactions (21). Our research revealed that IL-4, IL-5, IL-9, IL-10, and IL-13 were significantly increased in OT patients, indicating that Th2 cells may be the major effector T cells in OT. In addition, IL-5 showed the highest fold change in the OT group in our study. IL-5 induces terminal differentiation of activated B cells into antibody-forming cells in mice, promotes the proliferation and prolongs the survival of mature eosinophils (22). In cerebra of *Toxocara canis*-infected mice, Del Prete et al. also reported a prominent peak pattern of IL-5 (23). It is interesting to note that IL-5 and IL-13 were almost undetectable in non-OT uveitis in our study. These data are consistent with those of previous studies, which have shown that IL-5 is undetectable or mildly increased in non-infectious anterior uveitis and appears to be absent in panuveitis where there is a greater uveal tract involvement (13, 24). Thus, a significant increase of IL-5 and IL-13 in the AH of OT patients may propose further investigation of their role for OT pathogenesis.

On the other hand, our results revealed Th1 and Th17 cells may played minor role in OT pathogenesis. Out of 9 Th1 associated cytokines assigned in our study, 3 of them were undetectable among all three groups, including IL-2, IL-12p70, and TNF-β, while 4 of them were unchanged, including IL-7, IL-12p40, IL-15, and IFNR. Although TNF-α and IFN-α2 showed significant level increases, low expressions multiples at 2.4 and 1.3 times, respectively, were observed between OT and control group. Meanwhile, no Th1 cytokine showed significant difference between OT and non-OT uveitis group. These results suggest that Th1 cells may play mild role in OT pathogenesis. On the other hand, IL-17 was undetected among all groups. IL-17 is an essential proinflammatory cytokine for the host’s defense against bacteria and fungi, and its important role in autoimmune disease has only recently been discovered (25). Interestingly, our study demonstrated the absence of Th17 cells in OT inflammation. In addition, there were significant increases in TNF-α and IFN-γ (Th1) in non-OT uveitis group than that in controls, but there were no significant different in IL-17. Similarly, low concentration and no statistic difference of IL-17 expression were observed in patients with uveitis (26, 27). The role of IL-17 in uveitis will be interesting in further studies.

Interestingly, our results showed that IL-10 may be a crucial differentiating cytokine in OT. IL-6 and IL-10 were both detected in our study. IL-6 was considered a general inflammation marker in OT. In proinflammatory cytokines, IL-6 increases approximately 13-fold in OT compared with control group, however, our study showed almost the same expression in OT and non-OT uveitis groups. Elevated intraocular levels of IL-6 were found repeatedly in uveitis of diverse etiology (including ocular toxoplasmosis, viral uveitis, Fuchs uveitis syndrome, and Behcet’s uveitis), as well as in ocular fluids of children with uveitis (28, 29). Thus, IL-6 is considered a general marker of active uveitis and is not specific for particular uveitis entities (30, 31). This helps explain the similar expression level of IL-6 in both OT and uveitis patients in our study. In contrast, IL-10 is known as an anti-inflammatory cytokine that suppresses the expression of proinflammatory cytokines, including TNF-α, IFN-γ, and IL-1β (32). IL-10/IL-6 ratio has been assessed in lymphoma. In current study, IL-10/IL-6 ratio was less than 1.0 in all groups, with no statistic difference (*P* = 0.133).

The intraocular level of IL-10 reported in uveitis is still debatable (33–35). Elevated IL-10 level was associated with uveitis, but lower or no different IL-10 expressions, were also documented in uveitis. However, in our study, IL-10 showed significant difference in pairwise comparison between the three groups. It increased by 20-fold in OT, compared with controls, and 8-fold compared with the level in AH in non-OT uveitis eyes. Furthermore, TNF-α, IFN-γ, and IL-1β, whose expressions could be suppressed by elevated IL-10, were low-leveled or absent in our study (2.04, 1.39 and 1.34 ng/ml, respectively). These data suggest that IL-10 may be used as a unique biomarker to differentiate OT from non-OT uveitis and healthy subjects.

Our study did have some limitations. First, the study was conducted on a restricted small number of participants. Since OT is a rare disease and has been defined as a neglected parasitic infection, we are working on gathering more cases. Second, healthy people are actually the best control group; however, it is inappropriate to collect their AH by undertaking invasive procedures. Third, in our study, aqueous humor, but not vitreous samples, was collected. Vitreous might be able to provide more cytokines. However, aqueous humor paracentesis is faster and less invasive than AH sampling (36, 37). At last, the mean age of the patients in uveitis or cataract groups was higher than that of the OT group. However, no significant correlation was found between the AH cytokine levels and age, based on most previous studies (27).

## Conclusion

In conclusion, the cytokine profile expression in aqueous humor from patients with ocular toxocariasis was investigated, and our findings suggest that Th1 and Th17 cytokine responses are not enhanced, whereas the cytokine status was polarized toward a Th2 response. Our findings also suggest the involvement of IL-5, IL-10, and IL-13 in the immunopathogenesis of ocular toxocariasis. Current study may provide better understand of the pathogenesis and diagnostic markers of OT.

## Data Availability Statement

The original contributions presented in the study are included in the article/supplementary material, further inquiries can be directed to the corresponding author/s.

## Ethics Statement

The studies involving human participants were reviewed and approved by Investigational Review Board of Zhongshan Ophthalmic Center, Sun Yat-sen University. Written informed consent to participate in this study was provided by the participants’ legal guardian/next of kin.

## Author Contributions

XYD conceived and designed the study. ZJ, SL, and TZ participated in data collection, laboratory analysis, and interpretation. ZJ and XHD analyzed the data and wrote the first draft of the manuscript. XYD and LS critically reviewed the manuscript. All authors approved the submitted version.

## Conflict of Interest

The authors declare that the research was conducted in the absence of any commercial or financial relationships that could be construed as a potential conflict of interest.

## Publisher’s Note

All claims expressed in this article are solely those of the authors and do not necessarily represent those of their affiliated organizations, or those of the publisher, the editors and the reviewers. Any product that may be evaluated in this article, or claim that may be made by its manufacturer, is not guaranteed or endorsed by the publisher.
